# Whole Transcriptome Sequencing and Differential Analysis of Testes in Pre- and Post-Sexual Maturity *Bactrian Camels* (*Camelus bactrianus*)

**DOI:** 10.3390/biology14091254

**Published:** 2025-09-12

**Authors:** Xiaokang Chang, Xinkui Yao, Jun Meng, Jianwen Wang, Yaqi Zeng, Linling Li, Wanlu Ren

**Affiliations:** 1 College of Animal Science, Xinjiang Agricultural University, Urumqi 830052, China; changxk312@126.com (X.C.); yxk61@126.com (X.Y.); junm86@sina.com (J.M.); wjw1262022@126.com (J.W.); xjauzengyaqi@163.com (Y.Z.); lilinling@xjau.edu.cn (L.L.); 2 Xinjiang Key Laboratory of Equine Breeding and Exercise Physiology, Urumqi 830052, China

**Keywords:** *Junggar Bactrian camel*, testis, whole-transcriptome, testicular development, spermatogenesis

## Abstract

Testicular development in male animals is a conserved and highly regulated biological process. Investigating the molecular mechanisms underlying testicular development in *Junggar Bactrian camels* is essential for gaining a deeper understanding of this process in the species. This study selected testicular tissue from the *Junggar Bactrian camel* at pre-sexual maturity (G3 group, *n* = 4, 3 years old) and post-sexual maturity (G5 group, *n* = 4, 5 years old) for whole transcriptome sequencing and bioinformatics analysis. We identified differentially expressed mRNA (DEmRNA), including *KPNA2* and *LRRC46*; differentially expressed LncRNA (DELncRNA), including *LOC123613926* and *LOC123613624*; and differentially expressed miRNA (DEmiRNA), including *eca-miR-196a* and *eca-miR-183*. Additionally, we also identified 87 currently unnamed DEmiRNAs, which are of practical value for future research on the *Junggar Bactrian camel* testicular development and spermatogenesis. GO and KEGG enrichment analyses showed that DERNA are mainly involved in functions and processes such as protein binding (MF), protein import into nucleus (BP), and extracellular space (CC), as well as signaling pathways such as Insulin, FoxO, MAPK, and PI3K-Akt. Subsequently, we predicted some DEmiRNAs and DELncRNAs association with DEmRNAs, and constructed the competitive endogenous RNA (ceRNA) regulatory network. Finally, we randomly selected 10 DERNAs for RT-qPCR validation, and the transcriptome results were consistent with the RT-qPCR results, indicating that the sequencing results were true and reliable. In conclusion, this study analyzed the differential expression of mRNA, LncRNA, and miRNA in *Junggar Bactrian camels* before and after sexual maturity, providing data references for future studies related to testicular development and spermatogenesis.

## Simple Summary

In this study, we used whole transcriptome sequencing combined with a bioinformatics analysis to examine the differential gene expression in the testicular tissues of *Junggar Bactrian camels* before and after sexual maturity, thereby uncovering the genetic changes associated with testicular maturation. Our results indicate that non-coding RNA, including LncRNA and miRNA, are extensively and actively expressed in the testes of *Bactrian camels*. Genes, such as *KPNA2*, *LRRC46*, *eca-miR-196a*, and *eca-miR-183*, play crucial roles in testicular development and spermatogenesis, which is consistent with observations in other mammalian species, including yaks and mice. Notably, several types of miRNA were identified in this species for the first time, offering valuable insights for future research on the reproductive traits in *Junggar Bactrian camels*. An RT-qPCR analysis further verified that the expression profiles of ten selected RNA were in agreement with the sequencing data. These results provide an initial experimental framework and theoretical basis for studies on testicular development in *Junggar Bactrian camels* and offer reference data to support future reproductive breeding.

## 1. Introduction

The *Bactrian camel* belongs to Camelidae and *Camelus* [1]. As a valuable biological resource, this species is mainly distributed in countries such as China, Kazakhstan, and Russia, and is also found in small numbers in countries such as Iran, Turkey, and India [2]. According to the 2022 statistics from the Food and Agriculture Organization of the United Nations, there are currently 1.1 million *Bactrian camels* worldwide, of which approximately 461,700 are in China (http://faostat.fao.org/ (accessed on 16 August 2025)). Studies have shown that most male *Bactrian camels* exhibit a long growth cycle and low reproductive performance [3]. The *Junggar Bactrian camel’s* sexual maturity age is 5–6 years [4,5], which is much higher than other livestock. such as pigs (5–8 months), cattle (10–18 months), horses and donkeys (18–24 months), and sheep and goats (6–10 months) [6,7,8,9,10]. These unique biological characteristics result in a lower reproductive efficiency in *Bactrian camels*, making an in-depth investigation into the patterns of testicular growth and development before and after sexual maturity crucial for enhancing their reproductive rate. Furthermore, exploring the molecular mechanism of the *Bactrian camel* testicular development through transcriptomics can provide critical molecular targets and a theoretical basis for the camel genetic resource conservation and population recovery.

The testis is an important component of the male animals’ reproductive system, and its main functions are to produce sperm and androgen [11]. In the male animals’ reproductive system, whole transcriptome sequencing technology has become a powerful tool for our in-depth study of reproductive development and regulation, among other aspects [12]. In recent years, researchers have performed transcriptome sequencing on various livestock testicular tissue to deeply investigate the molecular regulatory mechanism of testicular development. For example, La et al. [13] identified 30 DEmRNAs, 23 DELncRNAs, and 277 DEcircRNAs in the testes of 6-, 18-, and 30-month-old yak. Xi et al. [14] comprehensively analyzed the role of N6-methyladenosine in testicular development through whole transcriptome and m6A methylome analysis. Yang et al. [15] screened out the key genes that lead to the formation of yak unilateral cryptorchidism through the transcriptome and the proteome.

Data from the National Center for Biotechnology Information (NCBI) indicates that, during the period of 2024–2025 alone, there were 100 studies on the transcriptome sequencing of bovine testes, and many reports on pigs (119), sheep (66), and goats (54). However, there were only eight studies on the transcriptome sequencing of the *Bactrian camel* testes (https://www.ncbi.nlm.nih.gov/ (accessed on 16 August 2025)). The above data demonstrates that, to date, our understanding of the gene expression pattern in the *Junggar Bactrian camel* testicular tissue remains very limited.

This study systematically analyzed the expression profiles of mRNA, LncRNA, and miRNA in the testicular tissues of *Junggar Bactrian camels* before and after sexual maturity using whole transcriptome sequencing. We performed GO and KEGG pathway enrichment analyses on DERNA, constructed a competitive endogenous RNA (ceRNA) regulatory network, and validated the expression of key genes using RT-qPCR. This study not only provides a novel theoretical basis for investigating reproductive regulation in male *Junggar Bactrian camel*, but offers a valuable model for elucidating the molecular regulatory mechanism underlying mammalian testicular development and spermatogenesis.

## 2. Materials and Methods

### 2.1. Animals and Sample Collection

This study was approved by the Animal Welfare and Ethics Committee of Xinjiang Agricultural University (approval number: 2025001). In this study, eight *Junggar Bactrian camels* raised under identical conditions in the Tacheng area of Xinjiang were selected as the experimental animals. According to their sexual maturity stage, they were divided into two groups: a pre-sexual maturity group (Group G3, *n* = 4, 3 years old) and a post-sexual maturity group (Group G5, *n* = 4, 5 years old). The castrated testicular tissue collected from the left side of each *Bactrian camel* was promptly aliquoted and preserved: one portion was snap-frozen in liquid nitrogen, and the other was fixed in 4% paraformaldehyde solution, with all samples stored for future use.

### 2.2. Micromorphological Examination

Camel testicular tissue was fixed in 10% neutral buffered formalin, dehydrated using an ethanol gradient, cleared with xylene, embedded in paraffin, and sectioned at a thickness of 4–5 μm. Tissue sections were prepared according to a previously described method [16], stained with hematoxylin and eosin (H&E), and photographed under a light microscope (Eclipse E100, Nikon, Tokyo, Japan).

### 2.3. RNA Extraction, Quality, and Integrity Assessment

Total RNA was extracted using the TRIzol™ reagent kit (Invitrogen, Carlsbad, CA, USA). RNA quality was assessed using a Qubit 3.0 Fluorometer (Life Technologies, Carlsbad, CA, USA) and a 2100 Bioanalyzer (Agilent Technologies, Santa Clara, CA, USA). Following the quality assessment, two libraries were constructed: (1) a ribosome-depleted strand-specific library, in which rRNA was removed using a ribosome-depletion kit, the remaining RNA fragmented, and a strand-specific library generated using dUTP labeling; (2) an miRNA library, in which adapters were ligated to both ends of the RNA, followed by reverse transcription and PCR amplification, with PCR products purified by PAGE and the 140 bp fragments recovered. Both libraries were assessed for quality using the Agilent 2100 Bioanalyzer (Agilent Technologies, Inc., Santa Clara, CA, USA)and quantified by qPCR before sequencing on the Illumina platform. The sequencing output was 12 Gb for the LncRNA library and 10 M reads for the miRNA library, providing comprehensive transcriptome data covering mRNA, LncRNA, circRNA, and miRNA. Transcriptome sequencing was performed by Hangzhou Astrocyte Technology Co., Ltd. (Hangzhou, China) using the HiSeq X10 platform (Illumina, Inc., San Diego, CA, USA).

### 2.4. Quality Assessment, Alignment Optimization, and Transcriptome Reconstruction of RNA-Seq Data

To obtain high-quality, clean reads, raw sequencing reads were further filtered using FASTP v0.11.9. The filtering criteria included (1) removing reads containing adapter sequences, (2) removing reads with more than 10% unknown nucleotides (N), and (3) removing low-quality reads in which more than 50% of bases had a Q-value ≤ 20) [17]. A reference genome index was then constructed, and the paired-end clean reads were aligned to the reference genome using HISAT2 v2.2.1, with all other parameters set to default [18]. (Reference genome: NCBI GCF_000767855.1_Ca_bactrianus_MBC_1.0, Annotation Release 102, 20 December 2021.) StringTie v2.2.1 was then used to independently assemble the aligned reads against the reference genome. All transcripts were subsequently merged to generate a comprehensive dataset [19].

### 2.5. Identification of Differentially Expressed Genes

The coding potential of transcripts was assessed using CPC2 and Pfam. CPC2 v2.0 evaluates each transcript based on the open reading frame (ORF) length, codon usage, and sequence conservation to predict whether it is likely to encode a protein. Transcripts with a CPC2 score < 0 were classified as non-coding [20]. Using HMMER v3.3.2, transcripts were searched against the Pfam database (v35.0), and transcripts with no significant matches to known protein domains (E-value < 1 × 10^−5^) were considered non-coding. A novel transcript was designated as an LncRNA only if it met all of the following criteria: (1) length > 200 nt; (2) CPC2 score < 0; (3) no Pfam domain matches; (4) no overlap with known coding genes, as determined by BLAST v2.13.0 against RefSeq coding sequences (E-value < 1 × 10^−10^).

The miRNA annotation was performed using miRBase v22.1, the most comprehensive database of mature miRNA sequences and hairpin precursors. Specifically, (1) mature miRNA sequences were aligned to miRBase’s “mature.fa” (v22.1) using Bowtie2 v2.4.5; (2) only reads exhibiting 100% identity to mature miRNA in miRBase were retained for quantification; and (3) novel miRNA absent from the miRBase were predicted using miRDeep2 v2.0.1.3 and further validated by confirming their hairpin structures with RNAfold v2.4.18 and their presence in at least two independent replicates.

Gene expression levels were quantified using the featureCounts v2.0.3 tool in the Subread package, and transcript expression was quantified using StringTie v2.2.1. Fragments Per Kilobase per Million mapped fragments (FPKM) values were used to assess gene expression correlation within and between testicular samples. A differential expression analysis of testicular genes between the pre- and post-sexual maturity groups was conducted using DESeq2 v1.34.0, with a significance threshold of |log2FC| ≥ 1 and False Discovery Rate of (FDR) < 0.05.

This study applied ComBat (sva package v3.42.0) to correct for the sequencing batch effects, and the effectiveness of the correction was evaluated using the following methods: (1) ANOVA: the contribution of batch effects to total variance decreased from 18% (pre-correction) to 3% (post-correction); (2) PCA: after correction, PC1 (28% variance) separated biological groups, while PC3 (5% variance) no longer distinguished batches; (3) rRNA contamination: following ribosomal RNA depletion, rRNA reads accounted for less than 5% of the total reads, as assessed by HISAT2 alignment to the rRNA genes; and (4) 3′ bias in RNA-seq: calculated as the ratio of reads mapping to the 3′ versus 5′ ends of genes (median ratio = 1.2, with values < 2.0 indicating acceptable uniformity).

### 2.6. Functional Enrichment Analysis of Differentially Expressed Genes

GO and KEGG pathway enrichment analyses were conducted using the online tool KOBAS (analysis date: 21 April 2025), and pathways with *p*-value < 0.05 were considered significantly enriched. The enriched GO terms and KEGG pathways were visualized using the clusterProfiler package in R v3.42.0.

### 2.7. Constructed ceRNA Network

To construct a ceRNA regulatory network involving LncRNA, miRNA, and mRNA, this study was conducted according to the following steps: first, miRanda v3.3a was employed to predict the interactions between LncRNA and miRNA; second, miRDB and TargetScan v8.0 were used in combination to systematically predict the targeting regulatory relationships between miRNA and mRNA; finally, Cytoscape v3.6.0 was utilized to visually integrate the key interaction relationships, thereby systematically illustrating the regulatory roles of LncRNA-miRNA-mRNA within the ceRNA network.

### 2.8. Validation of Differentially Expressed Genes

Ten DEGs were randomly selected to validate the RNA-Seq results using reverse transcription quantitative PCR (RT-qPCR). Total RNA was reverse-transcribed into cDNA using the G3337 Reverse Transcription Kit (Wuhan Servicebio Technology Co., Ltd., Wuhan, China). βactin served as the reference gene, and primers were designed using Primer Premier 5.0; the sequences are listed in Table 1. RT-qPCR was carried out on a CFX Connect Real-Time PCR System (Bio-Rad, Hercules, CA, USA). Each 15 μL reaction contained 7.5 μL 2× Universal Blue SYBR Green qPCR Master Mix, 1.5 μL of each primer, 2.0 μL cDNA, and 4.0 μL nuclease-free water. The PCR program consisted of an initial denaturation at 95 °C for 30 s, followed by 40 cycles of 95 °C for 15 s and 60 °C for 30 s, with fluorescence signals recorded every 0.5 °C during the temperature ramp. Relative gene expression was calculated using the 2^−ΔΔCt^ method and log2-transformed. All reactions were performed in technical triplicate.

## 3. Results and Analysis

### 3.1. Histological Analysis of Testicular Tissues

**Figure 1:**
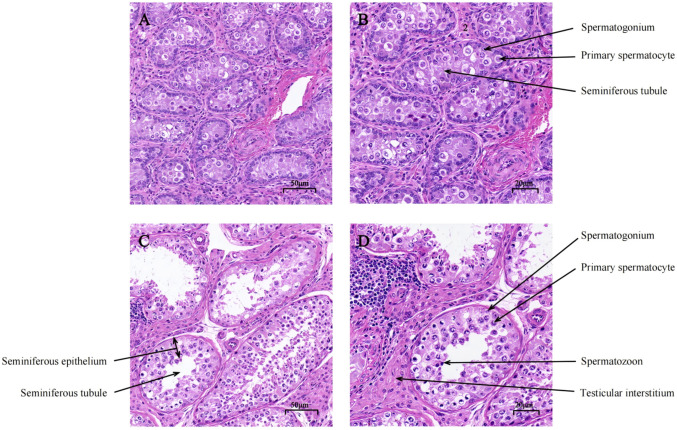
Morphological structure of testes at different developmental stages: (**A**) the morphological structure of pre-sexual maturity camel testes (400×); (**B**) the morphological structure of pre-sexual maturity camel testes (800×); (**C**) the morphological structure of post-sexual maturity camel testes (400×); (**D**) the morphological structure of post-sexual maturity camel testes (800×).

The morphological differences in testicular tissues of *Bactrian camels* at different developmental stages are shown in Figure 1. It is evident that, before sexual maturity, the testicular tissues are underdeveloped, with sparsely arranged seminiferous tubules and a relatively low number of spermatogenic cells and testicular interstitium (Figure 1A,B). After sexual maturity, the histological structure of the testis changes significantly, including an increased area of seminiferous tubules and thickness of the stratified seminiferous epithelium, along with the presence of a large number of mature sperm within the tubules (Figure 1C,D). These changes indicate that, during the process of sexual maturation, not only does the development of the seminiferous tubules accelerate, but the number of testicular interstitial cells also increases, all of which reflect the progressive maturation of testicular function.

### 3.2. RNA-Seq Data Analysis

A total of 16 cDNA libraries were constructed in this study. The testicular transcriptome generated approximately 970 million high-quality reads (an average of 60,949,049.4 reads per library). The GC content, Q20, and Q30 percentages of the reads ranged from 50.32% to 78.54%, 91.96% to 98.22%, and 83.07% to 96.83%, respectively. Moreover, more than 82.82% of the clean reads from the testicular tissues aligned with the reference genome (Table 2 and Table 3).

### 3.3. Gene Expression Quantification

**Figure 2:**
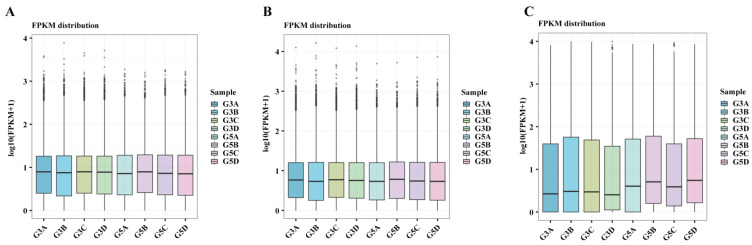
Comparative analysis of testicular transcriptome expression levels between G5 vs. G3: (**A**) distribution of mRNA expression levels; (**B**) distribution of LncRNA expression levels; (**C**) distribution of miRNA expression levels. In the figure, G3 represents the pre-sexual maturity stage, and G5 represents the post-sexual maturity stage. The same notation applies hereafter.

Boxplots were used to visualize the distribution of gene expression levels across the samples. The expression profiles of mRNA and LncRNA in testicular tissues of the pre- and post-sexual maturity groups are shown in Figure 2A,B. The variation in gene expression among the different samples was relatively small, indicating that the overall expression levels between immature and mature testicular samples were consistent. By contrast, miRNA expression showed a certain degree of variation, with testicular samples from sexually mature camels exhibiting slightly greater heterogeneity. This may be attributed to biological characteristics, as sexually mature testes are likely influenced by more complex regulatory factors (Figure 2C).

### 3.4. Correlation Analysis of Differentially Expressed Genes in Testicular Tissues at Different Developmental Stages

**Figure 3:**
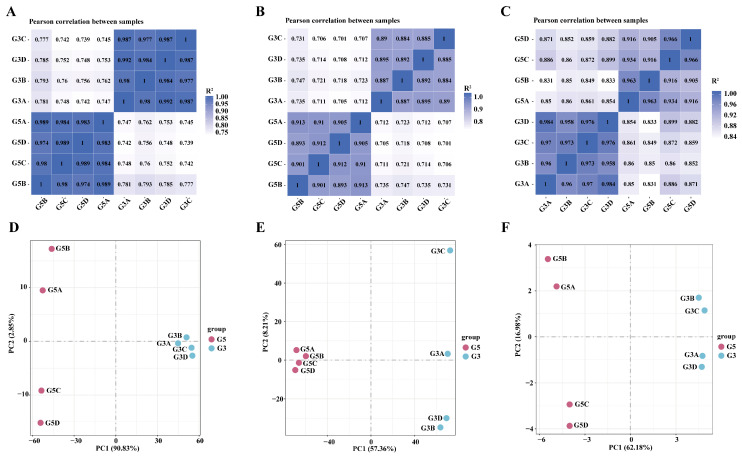
Correlation heatmap and principal component analysis (PCA) plot between G5 vs. G3: (**A**) correlation heatmap of mRNA between G5 and G3 samples; (**B**) correlation heatmap of LncRNA between G5 and G3 samples; (**C**) correlation heatmap of miRNA between G5 and G3 samples; (**D**) PCA plot of mRNA from G5 and G3 samples; (**E**) PCA plot of LncRNA from G5 and G3 samples; (**F**) PCA plot of miRNA from G5 and G3 samples.

Based on gene expression levels, we analyzed the correlation within and between groups by plotting Spearman correlation heatmaps and PCA plots. In Figure 3A–C, the Pearson correlation coefficients (R^2^) approached 1. By contrast, Figure 3D–F show greater dispersion between the pre- and post-maturity testicular samples, indicating high reproducibility of mRNA, LncRNA, and miRNA expression within each group and significant differences between the two groups.

### 3.5. Identification and Bioinformatics Analysis of Differentially Expressed mRNA

**Figure 4:**
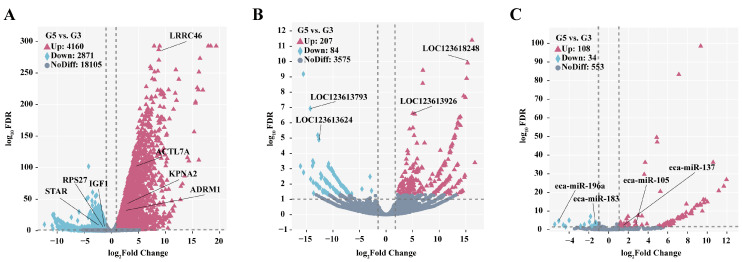
Volcano plot of DEGs between G5 vs. G3: (**A**) volcano plot of DEmRNA; (**B**) volcano plot of DELncRNA; (**C**) volcano plot of DEmiRNA. In the figure, “up” represents upregulated DEGs, “down” represents downregulated DEGs, and “NO Diff” indicates non-significantly differentially expressed genes.

**Figure 5:**
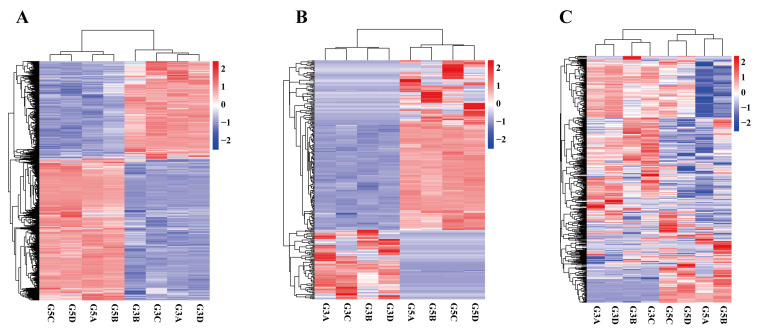
Clustering analysis of DEGs between G5 vs. G3: (**A**) cluster analysis of DEmRNA; (**B**) cluster analysis of DELncRNA; (**C**) cluster analysis of DEmiRNA. In the figure, the horizontal axis clusters samples with similar expression patterns, while the vertical axis groups genes based on expression profile similarity. Red indicates upregulated expression, and blue represents downregulated expression.

An analysis of testicular tissues from *Junggar Bactrian camels* before and after sexual maturity identified 7031 DEmRNAs, including key genes such as *KPNA2*, *ADRM1*, *LRRC46*, *RPS27*, *ACTL7A*, *IGF1*, and *STAR*. Among these, 4169 DEmRNAs were upregulated, such as *KPNA2* (log_2_FC = 2.72, FDR < 0.01), *ADRM1* (log_2_FC = 2.36, FDR < 0.01), *LRRC46* (log_2_FC = 8.78, FDR < 0.01), and *ACTL7A* (log_2_FC = 4.83, FDR < 0.01). By contrast, 2871 DEmRNAs were downregulated, including *RPS27* (log_2_FC = −1.80, FDR < 0.01), *STAR* (log_2_FC = 1.90, FDR < 0.01), and *IGF1* (log_2_FC = −1.03, FDR < 0.01) (Figure 4A and Table S1). A cluster analysis showed that DEmRNA displayed high reproducibility within groups but significant differences between groups (Figure 5A).

**Figure 6:**
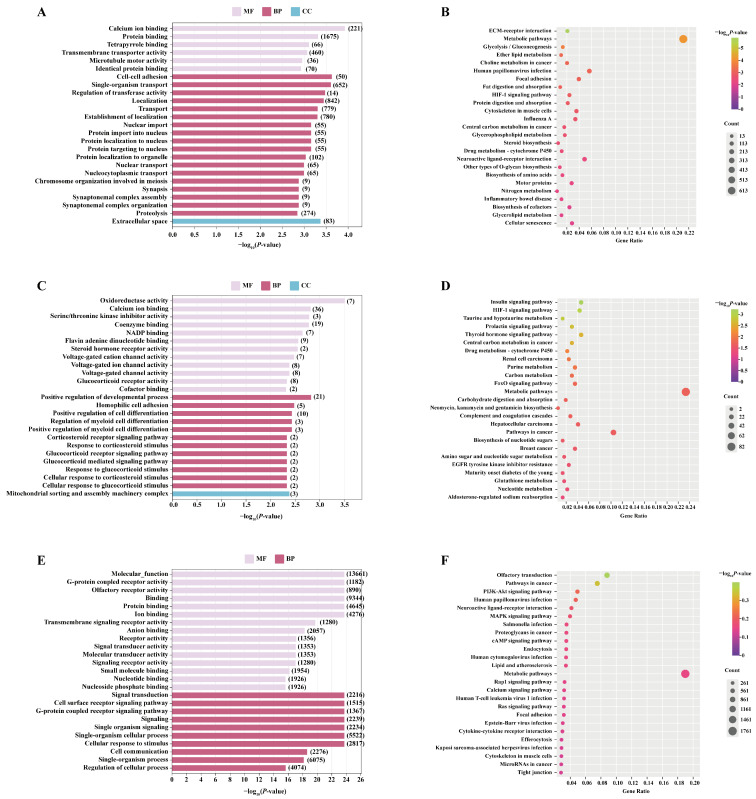
GO and KEGG enrichment analysis of DEGs in the G5 vs. G3: (**A**) GO annotation of DEmRNA; (**B**) KEGG enrichment of DEmRNA; (**C**) GO annotation of DELncRNA; (**D**) KEGG enrichment of DELncRNA; (**E**) GO annotation of DEmiRNA; (**F**) KEGG enrichment of DEmiRNA. In figures (**A**,**C**,**E**), the horizontal axis represents the significance of each term, with larger values indicating higher statistical significance. The number in parentheses denotes the number of genes enriched in the corresponding term. In figures (**B**,**D**,**F**), the circle color reflects the significance level of the pathway, while the circle size represents the number of genes enriched in that pathway.

A GO enrichment analysis revealed that DEmRNA was significantly enriched in terms related to cell adhesion (BP, *p* < 0.01), protein binding (MF, *p* = 0.00048144), protein import into nucleus (BP, *p* = 0.0006934), and extracellular space (CC, *p* = 0.00042621) (Figure 6A and Table S2). A KEGG pathway enrichment analysis indicated that DEmRNA was enriched in the ECM-receptor interaction (*p* < 0.01), metabolic pathways (*p* < 0.01), motor proteins (*p* = 0.0061), glycerophospholipid metabolism (*p* = 0.0026), and other pathways associated with testicular development and spermatogenesis (Figure 6B and Supplementary Table S2).

### 3.6. Characteristics of Differentially Expressed LncRNA

As shown in Figure 4B, a total of 291 DELncRNAs were identified, including *LOC123613926*, *LOC123613624*, *LOC123618248*, and *LOC123613793*. Among these, 207 DELncRNAs, such as *LOC123613926* (log_2_FC = 16.16, FDR < 0.01) and *LOC123618248* (log_2_FC = 15.33, FDR < 0.01), were upregulated, whereas 84 DELncRNAs, including *LOC123613624* (log_2_FC = −12.87, FDR < 0.01) and *LOC123613793* (log_2_FC = −14.27, FDR < 0.01), were downregulated. A clustering analysis revealed high reproducibility of DELncRNA expression within the same developmental stage, along with marked differences between the two stages (Figure 5B).

A GO enrichment analysis revealed that the target genes of these DELncRNAs were significantly enriched in 140 terms (*p* < 0.05) (Figure 6C and Table S2), including calcium ion binding (MF, *p* = 0.0015294), positive regulation of the developmental process (BP, *p* = 0.0014981), positive regulation of cell differentiation (BP, *p* = 0.0036957), and mitochondrial sorting and assembly machinery complex (CC, *p* = 0.0041396). A KEGG pathway analysis indicated that these target genes were primarily enriched in signaling pathways such as Insulin, HIF-1, and FoxO (*p* < 0.01) (Figure 6D and Table S2). These results suggest that LncRNA may play a critical role in regulating the genes associated with testicular development and spermatogenesis in *Junggar Bactrian camels*.

### 3.7. Screening of Differentially Expressed miRNA and Target Gene Enrichment Analysis

As shown in Figure 4C, a total of 142 DEmiRNAs were identified, including *eca-miR-105*, *eca-miR-137*, *eca-miR-196a*, and *eca-miR-183*. Of these, 108 DEmiRNAs, such as *eca-miR-105* (log_2_FC = 2.12, FDR = 0.028) and *eca-miR-137* (log_2_FC = 1.68, FDR = 0.018), were upregulated, while 34 DEmiRNAs, including *eca-miR-196a* (log_2_FC = −5.05, FDR < 0.01) and *eca-miR-183* (log_2_FC = −1.12, FDR < 0.004), were downregulated. Notably, 87 of the identified DEmiRNAs were previously unannotated, indicating that research on the miRNA of testicular tissue in *Bactrian camels* remains limited. As these novel miRNAs were identified through genome alignment and stringent criteria, they represent a valuable resource for future investigations into the molecular mechanisms governing testicular development and spermatogenesis in this species. A clustering analysis showed high reproducibility of DEmiRNA expression within each group and significant differences between the two groups (Figure 5C).

A GO enrichment analysis revealed that the target genes of these DEmiRNAs were significantly enriched in 140 terms (*p* < 0.05) (Figure 6E and Table S2). Representative examples included G-protein coupled receptor activity (MF, *p* < 0.01), ion binding (MF, *p* < 0.01), protein binding (MF, *p* < 0.01), and cell communication (BP, *p* < 0.01), all of which are associated with testicular development and spermatogenesis. A KEGG pathway analysis indicated that none of the 358 enriched pathways reached statistical significance (*p* > 0.05) (Figure 6F and Table S2), including the Rap1 signaling pathway (*p* = 0.718), the PI3K-Akt signaling pathway (*p* = 0.602), and the MAPK signaling pathway (*p* = 0.67).

### 3.8. LncRNA-miRNA-mRNA in the ceRNA Network

**Figure 7:**
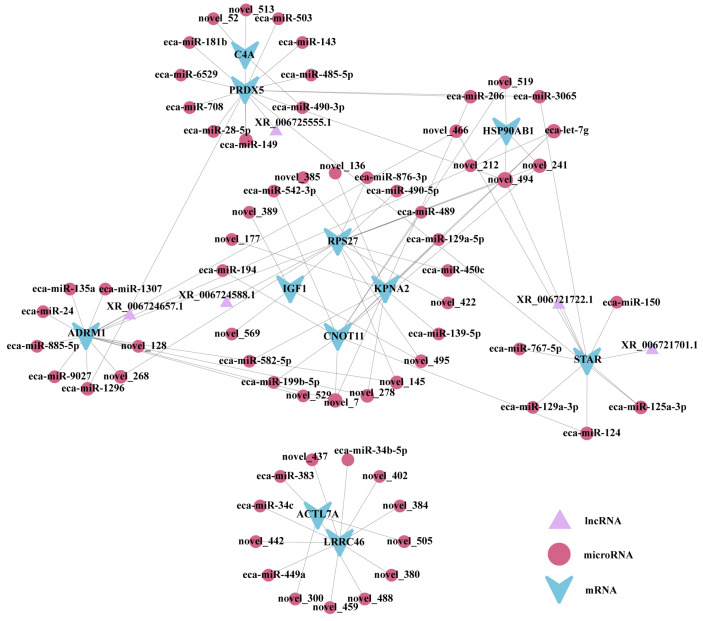
LncRNA-miRNA-mRNA in the ceRNA network.

By integrating the analysis results of the DEmiRNA-DEmRNA and the DELncRNA-DEmRNA interaction networks (Figure 7), this study successfully identified a set of DEmRNAs co-regulated by multiple miRNAs and LncRNAs, suggesting that these gene clusters may play a central regulatory role in testicular development and spermatogenesis.

### 3.9. Validation of RNA-Seq Results

**Figure 8:**
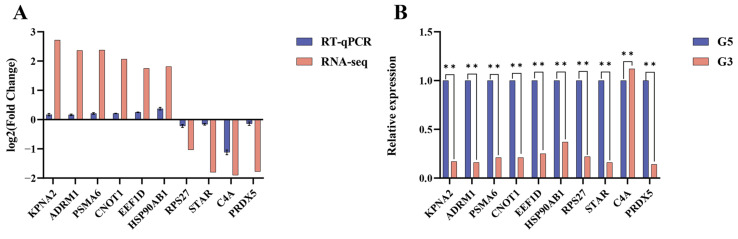
Validation of RNA-Seq results using RT-qPCR in *Bactrian camels*: (**A**) comparison of Log_2_fold change (Log_2_FC) of DEmRNA obtained from RNA-seq and RT-qPCR; (**B**) relative expression of DEmRNA measured by RT-qPCR. In Figure B, ** indicates a highly significant difference (*p* < 0.01).

To validate the RNA-Seq results, ten genes-*KPNA2*, *ADRM1*, *PSMA6*, *CNOT1*, *EEF1D*, *HSP90AB1*, *RPS27*, *STAR*, *C4A*, and *PRDX5*-were randomly selected for RT-qPCR analysis. The expression patterns observed by RT-qPCR were consistent with the RNA-Seq data, confirming the reliability of the sequencing results for downstream analyses (Figure 8A). Intergroup differences were assessed using Student’s *t*-test and one-way ANOVA in GraphPad Prism (v9.4.1), with significance set at * *p* < 0.05 and ** *p* < 0.01. Validation of the DEGs in testicular tissues before and after maturity indicated that C4A exhibited the greatest upregulation before sexual maturity (*p* < 0.01), while *KPNA2*, *ADRM1*, PSMA6, *CNOT1*, *EEF1D*, *HSP90AB1*, *RPS27*, *STAR*, and *PRDX5* exhibited the most significant downregulation (*p* < 0.01) (Figure 8B).

## 4. Discussion

The testis, as a vital reproductive organ, is responsible for the production, storage, and maturation of sperm, as well as the lifelong synthesis and secretion of androgens. These processes require coordination among different testicular cell types and involve tightly regulated and complex interactions [21,22]. The tissue morphology of the testis changes significantly before and after sexual maturity, primarily reflected in the structure of seminiferous tubules, the development of spermatogenic cells, and the function of testicular interstitial cells. A hallmark of testicular development is its extensive potential for proliferation and differentiation, particularly during puberty, which represents an ideal stage for investigating testicular development [23]. During puberty, the testes grow rapidly, primarily due to the expansion of germ cells and the androgen-induced increase in seminiferous tubule diameter [24]. Our observations of the testes are consistent with previous findings [25], suggesting that age has a significant effect on the histoanatomical structure of the camel testis.

Testicular development is a critical factor determining fertility in camels, with gene expression regulation playing a central role in both testis development and spermatogenesis [26]. Previous studies have shown that male mice lacking *KPNA2* exhibit reduced body size and sperm motility, increased sperm abnormalities, and disrupted testicular gene expression, ultimately leading to infertility [27]. Similarly, Li et al. [28] reported that significant downregulation of *HERC4* in mouse testes induces comparable phenotypic effects. In this study, the expression levels of *KPNA2* and *HERC4* in testicular tissues of sexually mature *Bactrian camels* were significantly higher than those in pre-sexual maturity individuals. A comparative analysis with mice suggests that *KPNA2* and *HERC4* are highly conserved and play essential roles in sexual maturation and testicular development. These genes likely facilitate nucleocytoplasmic transport of testis-specific transcription factors in seminiferous tubules and influence spermatogenesis by regulating the expression of extracellular matrix-related genes. They are enriched in the biological processes associated with testicular development and spermatogenesis, including protein binding and the positive regulation of cell differentiation.

Wu et al. [29] reported that insulin-like growth factor 1 (IGF1) may regulate the proliferation and differentiation of Leydig cells in Tibetan sheep. Similarly, the IGF1/IGF1R-mediated PI3K-Akt pathway has been shown to play a crucial role in maintaining the pluripotency of spermatogonial stem cells (SSCs) in mice [30]. Studies on Sunite *Bactrian camels* have further demonstrated that the expression of testicular *IGF1* increases progressively with age and during pubertal development [26]. These findings suggest that *IGF1* is a key regulator of testicular development across species. While its expression patterns and molecular mechanisms appear to be evolutionarily conserved, species-specific regulatory modes may also exist. Thus, clarifying the precise function of *IGF1* in the *Bactrian camel* testes is essential for understanding its unique role in reproductive biology in camels.

During testicular development, LncRNA acts as key regulatory factor that exerts essential functions by modulating critical biological processes, including testicular cell proliferation and differentiation, hormone synthesis and release, as well as spermatogenesis [31,32]. The expression profiles of LncRNA in testicular tissue have been identified and reported in several animal species, such as the mouse [33], the pig [34], cattle [35], and the horse [36]. However, only one study to date has examined LncRNA in camel testes [26]. Thus, comprehensive investigations of LncRNA in *Bactrian camels* and other camelids are urgently needed to address this major gap in regulatory research. Consistent with observations in the testes of other species, the KEGG enrichment analysis in this study revealed significant enrichment of the insulin signaling pathway. This pathway is primarily activated by insulin and IGF stimulation and regulates glucose metabolism, cell proliferation, differentiation, and survival, thereby maintaining metabolic homeostasis and energy balance [37]. Notably, unlike previous studies, we also found that *IGF1* was enriched as a LncRNA target gene in the FoxO signaling pathway, which plays a central role in cellular homeostasis, stress response, metabolism, and apoptosis [38]. Previous research has demonstrated that the insulin signaling pathway is essential for regulating testicular development, microenvironmental stability, and spermatogenesis [39]. Based on these findings, we propose that *IGF1* may contribute not only to the regulation of the insulin signaling pathway but to the modulation of the FoxO signaling pathway.

In animals, miRNA plays a key regulatory role in multiple physiological processes, including cell proliferation, metabolic homeostasis, and reproductive function [40]. Numerous studies have demonstrated that miRNA is essential for testicular development and spermatogenesis in humans [41], pigs [42], and cattle [43]. Studies in mice and pigs have shown that *miR-196a* and *miR-183* regulate reproductive performance by promoting the proliferation of Sertoli cells, inhibiting their apoptosis, and supporting spermatogenesis [44,45]. In this study, the DEmiRNAs, specifically *eca-miR-196a* and *eca-miR-183*, exhibited significant changes in expression before and after sexual maturity. These results indicate that *eca-miR-196a* and *eca-miR-183* may be involved in the regulation of testicular development in *Bactrian camels*, although their precise functions require further investigation and validation through in vitro experiments.

Due to interspecies differences in genetic and regulatory contexts, miRNA expression profiles in testicular tissues exhibit variation across animals. For example, Tang et al. [46] reported that *miR-9-5p* in mouse testes regulates the expression of its target, *SIRT1* mRNA, thereby modulating the activation of the FoxO signaling pathway. However, in the DEmiRNA and DEmRNA identified in this study, neither *miR-9-5p* nor *SIRT1* was detected. A comparative analysis with yak data revealed that *bta-miR-339b* and *novel32_star* play important and regulatory roles in the PI3K-Akt and MAPK signaling pathways, influencing the expression of multiple genes involved in testicular development and spermatogenesis [47]. Although the KEGG enrichment analysis in this study also indicated significant enrichment of the PI3K-Akt and MAPK pathways, *bta-miR-339b* and *novel32_star* were not identified. These results further suggest that miRNA-mRNA interactions regulating testicular development and spermatogenesis may be highly species-specific.

The ceRNA network in the *Bactrian camel* testes was constructed by integrating predicted regulatory interactions among the DELncRNA, DEmiRNA, and DEmRNA. Previous studies have shown that *miR-383* is significantly downregulated in the testes of men with spermatogenic arrest and infertility [48]. In this study, the expression of *eca-miR-383* in post-sexual maturity testes was significantly higher than in pre-sexual maturity individuals, thus further indicating the crucial role of miRNA in the reproductive regulation of male camels. Zhou et al. [49] reported that knockout of the highly expressed *ACTL7A* gene in mouse testes disrupts acrosome formation, causing male infertility and early embryonic developmental arrest. A target prediction suggested that *eca-miR-383* may regulate *ACTL7A*, implying that it could modulate spermatogenesis in camels through this target.

Yin et al. [50] demonstrated that *LRRC46* is a critical gene for flagellum formation and plays a key role in spermatogenesis and male fertility. In this study, *LRRC46* expression was low in the testes from *Bactrian camels* before sexual maturity but increased significantly after sexual maturity, further highlighting its essential role in testicular development and spermatogenesis. A bioinformatic analysis predicted that *eca-miR-34b-5p* may target *LRRC46*, contributing to the fine regulation of reproductive function in camels. However, the precise molecular mechanisms and biological functions of the *eca-miR-383-ACTL7A* and *eca-miR-34b-5p-LRRC46* axes in regulating testicular development and spermatogenesis in *Bactrian camels* remain unclear. Notably, the ceRNA regulatory network identified several novel DEmiRNA-DEmRNA pairs (e.g., *novel_136-KPNA2* and *novel_389-KPNA2*), providing valuable focal points for uncovering key miRNA and the regulatory targets involved in the testicular development of *Bactrian camels* and warranting further functional validation and mechanistic studies.

Although this study provides novel insights into the transcriptomic regulation of testicular development in *Bactrian camels*, several limitations should be noted. First, each group contained only four biological replicates (*n* = 4), resulting in a small sample size that may reduce statistical power and limit the comprehensive assessment of individual variation. Second, due to the absence of established camelid testicular cell lines, functional validation of candidate genes (e.g., *KPNA2*, *LRRC46*) and their regulatory interactions (e.g., *eca-miR-383-ACTL7A*) relied solely on bioinformatic predictions and has not yet been confirmed through in vivo or in vitro experiments. To address these limitations, future studies will expand the sample size to include individuals of varying ages and physiological states, thereby improving the reliability of the findings. Furthermore, primary testicular cell cultures and testicular organoid models for *Junggar Bactrian camels* will be established to provide an in vitro platform for functional studies. Using these systems, loss- and gain-of-function experiments targeting key signaling pathways (e.g., PI3K-Akt, FoxO, MAPK) and candidate genes will be conducted to clarify the precise regulatory mechanisms underlying sexual maturation and testicular development.

## 5. Conclusions

This study is the first to employ whole-transcriptome sequencing combined with bioinformatic analyses to systematically characterize the expression dynamics of mRNA, LncRNA, and miRNA during testicular development in *Junggar Bactrian camels* before and after sexual maturation. Notably, non-coding RNA such as LncRNA and miRNA are broadly and actively expressed in the camel testes. Compared with the known camel miRNA, we identified 87 novel DEmiRNAs, which not only facilitate the identification of key miRNA regulators of testicular development but expand the *Bactrian camel* miRBase database. RT-qPCR validation confirmed that the expression trends observed in the transcriptome data were consistent, supporting the reliability of the sequencing results. Several signaling pathways closely associated with testicular development and spermatogenesis-including the Insulin, FoxO, PI3K-Akt, and MAPK pathways-along with key genes, such as *KPNA2*, *HERC4*, and *IGF1*, were identified. These genes may participate in molecular interaction networks, including *eca-miR-383-ACTL7A* and *eca-miR-34b-5p-LRRC46*. Overall, this study provides a foundational framework for further investigating the functional roles of non-coding RNA in testicular development and spermatogenesis in male *Bactrian camels*, which requires subsequent in vitro functional experiments to elucidate the underlying molecular mechanisms.

## Figures and Tables

**Table 1:** The primers used for RT-qPCR (F = forward, R = reverse).

Gene	Accession Number	Primer Sequence (5′ → 3′)	Product Length (bp)
β-actin	XM_010965866.2	F: TCACTCACACTGTGCCCATC	276
		R: CGATGGTGATGACCTGACCG	
KPNA2	XM_010948667.2	F: TTGAAGAATGTGGAGGTTTGGA	231
		R: AACAAACAACTTTACGCCTCAGCT	
ADRM1	XM_045516234.1	F: CGACGACTCCCTCATTCACTTT	216
		R: ACCTTCCGGCAGTGTTCCTC	
PSMA6	XM_010967859.2	F: TTGACCGCCACATTACCATT	274
		R: TTAGCTGCCTCATAGCGTGCC	
CNOT1	XM_010967251.2	F: TGAAAGCACCACCAGAGGATGA	145
		R: TCTGGACAGTGTTTGATAGGGAAG	
HSP90AB1	XM_045509278.1	F: GATGCCTTGGACAAGATTCGTT	250
		R: CAACACCAAACTGCCCAATCA	
EEF1D	XM_010961152.2	F: CAAGTACGACGATGCAGAAAGGA	139
		R: AGCGAGGGACTTCTGGATGTT	
RPS27	XM_010954019.2	F: GGAGGATTTCCGCTTTCGCT	248
		R: CTGGCAGAGGACAGTAGAGC	
STAR	XM_045509996.1	F: AGACGTGGGCAAGGTGTTCC	116
		R: CCTTGACATTCGGATTCCACTC	
C4A	XM_010961471.2	F: GCGGAAGAAGGAGGTGTATGCT	134
		R: ACTGGGTGCTGCTGTTGGAAT	
PRDX5	XM_045518137.1	F: ACGGAAGGCAAGGTTCAGCT	163
		R: GCTCCACATTCAAGGACTTCACTA	

**Table 2:** Summary of mRNA and LncRNA analyses following reference genome alignment.

Sample	Raw Reads	Clean Reads	Q20	Q30	GC Content	Mapped Reads
G3A	107,793,310	107,593,776	97.98%	94.26%	51.53%	84,447,363 (78.49%)
G3B	106,556,608	106,350,854	97.91%	93.96%	52.56%	80,037,945 (75.26%)
G3C	104,963,176	104,776,860	98.01%	94.37%	50.86%	82,017,719 (78.28%)
G3D	105,845,590	105,667,472	97.99%	94.26%	50.99%	83,238,145 (78.77%)
G5A	106,950,304	106,752,926	98.00%	94.53%	52.11%	83,811,285 (78.51%)
G5B	105,116,432	104,942,932	98.22%	94.83%	51.76%	83,279,099 (79.36%)
G5C	104,563,528	104,384,630	98.01%	94.45%	51.09%	83,145,960 (79.65%)
G5D	107,799,328	107,629,916	98.21%	94.72%	50.32%	87,684,364 (81.47%)

**Table 3:** Summary of miRNA analyses following reference genome alignment.

Sample	Raw Reads	Clean Reads	Q20	Q30	GC Content	Mapped Reads
G3A	16,926,078	15,008,051	97.29%	93.32%	77.75%	12,583,375 (83.84%)
G3B	15,945,398	12,588,361	92.78%	85.45%	77.91%	11,070,688 (87.94%)
G3C	16,923,305	15,112,777	95.52%	90.17%	77.26%	13,308,521 (88.06%)
G3D	16,930,364	14,851,069	96.82%	92.19%	77.96%	13,521,818 (91.05%)
G5A	12,995,234	10,307,133	93.79%	86.86%	78.54%	86,165,400 (83.60%)
G5B	15,946,548	13,550,212	95.43%	88.71%	78.06%	11,290,231 (83.32%)
G5C	16,924,183	15,521,055	91.96%	83.07%	76.42%	13,735,688 (88.50%)
G5D	13,005,404	10,033,266	97.66%	93.71%	78.34%	8,979,809 (89.50%)

## Data Availability

The transcriptome data generated from this study have been deposited at NCBI BioProject (PRJNA1249912). In this study, differentially expressed mRNA, LncRNA, miRNA (Table S1), and GO and KEGG enrichment information of differentially expressed mRNA, LncRNA, and miRNA (Table S2) have been deposited at Figshare and are available at: https://doi.org/10.6084/m9.figshare.28838192.

## Supplementary Materials

The following supporting information can be downloaded at: https://www.mdpi.com/article/10.3390/biology14091254/s1, Table S1: Differentially expressed mRNAs, LncRNAs and miRNAs; Table S2: GO and KEGG enrichment information of differentially expressed mRNAs, LncRNAs, and miRNAs.

## References

[1] Si R., Ming L., Yun X., He J., Yi L., Na Q., Ji R., Dong T. (2025). Proteomics integrated with metabolomics: Analysis of the internal mechanism underlying changes in meat quality in different muscles from *Bactrian camels*. Food Chem. X.

[2] Burger P.A., Ciani E., Faye B. (2019). Old World camels in a modern world-a balancing act between conservation and genetic improvement. Anim. Genet..

[3] Yang D., Yuan L., Ma X., Qi Y., Cheng S., Zhang Y. (2024). Histological study of *Bactrian camel* cryptorchidism and expression of immunoglobulin λ light chain in the testicular and epididymis of cryptorchid *Bactrian camel*. Reprod. Domest. Anim..

[4] Yao H., Liang X., Dou Z., Zhao Z., Ma W., Hao Z., Yang J. (2023). Transcriptome analysis to identify candidate genes related to mammary gland development of *Bactrian camel* (*Camelus bactrianus*). Front. Vet. Sci..

[5] Hasi G., Wu L., Sodnompil T., Yi R., Wu R., Zhang R., Nasenochir N. (2023). Differential expression and localisation of heat shock protein 70 (HSP70) and glutathione peroxidase 5 (GPX5) in the testis and epididymis of Sonid *Bactrian camels*. Reprod. Fertil. Dev..

[6] Li X.L., Wu Q., Wang T., Zhang L., Wu X., Zhang Y., Liu B. (2023). Integrated testis transcriptome and whole genome analysis of sexual maturity in Large White and Tongcheng pigs. Reprod. Domest. Anim..

[7] Rawlings N., Evans A.C.O., Chandolia R.K., Bagu E.T. (2008). Sexual maturation in the bull. Reprod. Domest. Anim..

[8] Ebel F., Ulloa O., Strobel P., Ramírez-Reveco A. (2021). Semen quality and freezability analyses in the ejaculates of two Poitou donkeys in the southern hemisphere. Front. Vet. Sci..

[9] Zhang F.L., Zhang X.Y., Zhao J.X., Zhu K.X., Liu S.Q., Zhang T., Shen W. (2022). Multispecies comparative analysis reveals transcriptional specificity during Mongolian horse testicular development. Reprod. Domest. Anim..

[10] Zhao W., Adjei M., Zhang Z., Yuan Z., Cisang Z., Song T. (2023). The role of GnRH in Tibetan male sheep and goat reproduction. Reprod. Domest. Anim..

[11] Su J., Yang Y., Wang D., Su H., Zhao F., Zhang C., Zhang M., Li X., He T., Li X. (2025). A dynamic transcriptional cell atlas of testes development after birth in Hu sheep. BMC Biol..

[12] Słowińska M., Paukszto Ł., Jastrzębski J.P., Bukowska J., Kozłowski K., Jankowski J., Ciereszko A. (2020). Transcriptome analysis of turkey (*Meleagris gallopavo*) reproductive tract revealed key pathways regulating spermatogenesis and post-testicular sperm maturation. Poult. Sci..

[13] La Y., Ma X., Bao P., Chu M., Yan P., Liang C., Guo X. (2023). Genome-wide Landscape of mRNAs, LncRNAs, and circRNAs during Testicular Development of Yak. Int. J. Mol. Sci..

[14] Xi B., Lu Z., Zhang R., Zhao S., Li J., An X., Yue Y. (2025). Comprehensive analysis of the transcriptome-wide m6A Methylome in sheep testicular development. Genomics.

[15] Yang D., Yuan L., Zeng J., Qi Y., Ma L., Li H., Lv J., Chen Y. (2025). Comparative proteomic and transcriptomic analysis of testicular tissue of yaks with or without cryptorchidism. Theriogenology.

[16] Yao H., Liu M., Ma W., Yue H., Su Z., Song R., Ma Q., Li L., Wu Z., Ma Y. (2022). Prevalence and pathology of *Cephalopina titillator* infestation in *Camelus bactrianus* from Xinjiang, China. BMC Vet. Res..

[17] Chen S., Zhou Y., Chen Y., Gu J. (2018). fastp: An ultra-fast all-in-one FASTQ preprocessor. Bioinformatics.

[18] Pertea M., Kim D., Pertea G.M., Leek J.T., Salzberg S.L. (2016). Transcript-level expression analysis of RNA-seq experiments with HISAT, StringTie and Ballgown. Nat. Protoc..

[19] Pertea M., Pertea G.M., Antonescu C.M., Chang T.-C., Mendell J.T., Salzberg S.L. (2015). StringTie enables improved reconstruction of a transcriptome from RNA-seq reads. Nat. Biotechnol..

[20] Kong L., Zhang Y., Ye Z.-Q., Liu X.-Q., Zhao S.-Q., Wei L., Gao G. (2007). CPC: Assess the protein-coding potential of transcripts using sequence features and support vector machine. Nucleic Acids Res..

[21] Lara N.d.L.e.M., Sakib S., Dobrinski I. (2021). Regulation of cell types within testicular organoids. Endocrinology.

[22] Oliver E., Stukenborg J.B. (2020). Rebuilding the human testis in vitro. Andrology.

[23] Luo L., Sun L., Li S., Liu H., Chen Z., Huang S., Mo Y., Li G. (2024). miR-124-3p regulates the involvement of *Ptpn1* in testicular development and spermatogenesis in mouse. Gene.

[24] Koskenniemi J.J., Virtanen H.E., Toppari J. (2017). Testicular growth and development in puberty. Curr. Opin. Endocrinol. Diabetes Obes..

[25] Contreras-Ortiz A.J., Vigueras R.M., Mendoza-Elvira S.E., Martínez-Castañeda F.E., Gutiérrez-Pérez O., Trujillo-Ortega M.E. (2021). Postnatal testicular development in Vietnamese pot-bellied pigs. Acta Histochem..

[26] Hasi G., Sodnompil T., Na H., Liu H., Ji M., Xie W., Nasenochir N. (2023). Whole transcriptome sequencing reveals core genes related to spermatogenesis in *Bactrian camels*. J. Anim. Sci..

[27] Navarrete-López P., Maroto M., Pericuesta E., Fernández-González R., Lombó M., Ramos-Ibeas P., Gutiérrez-Adán A. (2023). Loss of the importin *Kpna2* causes infertility in male mice by disrupting the translocation of testis-specific transcription factors. Iscience.

[28] Li H., Yang B.-Y., Liu M.-M., Zhao S.-W., Xie S.-Z., Wang H., Zhang S., Xuan X.-N., Jia L.-J. (2021). Reproductive injury in male BALB/c mice infected with *Neospora caninum*. Parasites Vectors.

[29] Wu Y., Li Q., Qi X., Liu Z., Wang C., Zhao X., Ma Y. (2024). Molecular characteristics and regulatory role of insulin-like growth factor 1 gene in testicular Leydig cells of Tibetan sheep. Sci. Rep..

[30] Huang Y.H., Chin C.C., Ho H.N., Chou C.K., Shen C.N., Kuo H.C., Ling T.Y. (2009). Pluripotency of mouse spermatogonial stem cells maintained by IGF-1-dependent pathway. FASEB J..

[31] Wang J., Ren Q., Hua L., Chen J., Zhang J., Bai H., Li H., Xu B., Shi Z., Cao H. (2019). Comprehensive analysis of differentially expressed mRNA, LncRNA and circRNA and their ceRNA networks in the Longissimus Dorsi muscle of two different pig breeds. Int. J. Mol. Sci..

[32] Yan Q., Wang Q. (2025). Exploring the characters of non-coding RNAs in spermatogenesis and male infertility. Int. J. Mol. Sci..

[33] Han G., Hong S.H., Lee S.J. (2021). Transcriptome analysis of testicular aging in mice. Cells.

[34] Ran M., Chen B., Li Z., Wu M., Liu X., He C., Li Z. (2016). Systematic identification of long noncoding RNAs in immature and mature porcine testes. Biol. Reprod..

[35] Gao Y., Li S., Lai Z., Zhou Z., Wu F., Huang Y., Dang R. (2019). Analysis of long non-coding RNA and mRNA expression profiling in immature and mature bovine (*Bos taurus*) testes. Front. Genet..

[36] Liu Y., Du M., Zhang L., Wang N., He Q., Cao J., Dugarjaviin M. (2024). Comparative analysis of mRNA and LncRNA expression profiles in testicular tissue of sexually immature and sexually mature mongolian horses. Animals.

[37] Sakaguchi M. (2024). The role of insulin signaling with FOXO and FOXK transcription factors. Endocr. J..

[38] Liu W., Meng P., Li Z., Shen Y., Meng X., Hu M. (2025). The multifaceted impact of physical exercise on FoxO signaling pathways. Front. Cell Dev. Biol..

[39] Yildirim O.G., Guney C., Alcigir M.E., Akar F. (2023). High-fructose consumption suppresses insulin signaling pathway accompanied by activation of macrophage and apoptotic markers in rat testis. Reprod. Biol..

[40] Ling Y.H., Ren C.H., Guo X.F., Xu L.N., Huang Y.F., Luo J.C., Zhang Y.H., Zhang X.R., Zhang Z.J. (2014). Identification and characterization of microRNAs in the ovaries of multiple and uniparous goats (*Capra hircus*) during follicular phase. BMC Genom..

[41] Chen W., Cui Y., Ning M., Zhang H., Yin C., He Z. (2022). The mechanisms and functions of microRNAs in mediating the fate determinations of human spermatogonial stem cells and Sertoli cells. Semin. Cell Dev. Biol..

[42] Zhang B., Yan Z., Gao Y., Li J., Wang Z., Wang P., Gun S. (2022). Integrated analysis of miRNA and mRNA expression profiles in testes of Landrace and Hezuo boars. Front. Vet. Sci..

[43] Wu C., Blondin P., Vigneault C., Labrecque R., Sirard M.A. (2020). Sperm miRNAs-Potential mediators of bull age and early embryo development. BMC Genom..

[44] Zhou L., Su X., Li B., Chu C., Sun H., Zhang N., Han B., Li C., Zou B., Niu Y. (2019). PM2.5 exposure impairs sperm quality through testicular damage dependent on NALP3 inflammasome and miR-183/96/182 cluster targeting FOXO1 in mouse. Ecotoxicol. Environ. Saf..

[45] Zhang S., Guo J., Liang M., Qi J., Wang Z., Jian X., Zhang Z., Sun B., Li Z. (2019). miR-196a Promotes Proliferation and Inhibits Apoptosis of Immature Porcine Sertoli Cells. DNA Cell Biol..

[46] Tang Y., Sun L., Li S., Luo L., Liu H., Chen Z., Li G. (2024). miR-9-5p regulates Sirt1 involved in testicular development and spermatogenesis in mouse. Theriogenology.

[47] La Y., Ma X., Bao P., Chu M., Guo X., Liang C., Yan P. (2023). Identification and profiling of microRNAs during yak’s testicular development. BMC Vet. Res..

[48] Tian H., Cao Y.X., Zhang X.S., Liao W.P., Yi Y.H., Lian J., Sun F. (2013). The targeting and functions of miRNA-383 are mediated by FMRP during spermatogenesis. Cell Death Dis..

[49] Zhou X., Liu Z., Jia W., Hou M., Zhang X. (2022). *Actl7a* deficiency in mice leads to male infertility and fertilization failure. Biochem. Biophys. Res. Commun..

[50] Yin Y., Mu W., Yu X., Wang Z., Xu K., Wu X., Cai Y., Zhang M., Lu G., Chan W.Y. (2022). LRRC46 accumulates at the midpiece of sperm flagella and is essential for spermiogenesis and male fertility in mouse. Int. J. Mol. Sci..

